# Predictors of Treatment Failure and Mortality among Patients with Septic Shock Treated with Meropenem in the Intensive Care Unit

**DOI:** 10.21315/mjms2024.31.1.7

**Published:** 2024-02-28

**Authors:** Mohd Zulfakar Mazlan, Amar Ghassani Ghazali, Mahamarowi Omar, Najib Majdi Yaacob, Nik Abdullah Nik Mohamad, Mohamad Hasyizan Hassan, Wan Fadzlina Wan Muhd Shukeri

**Affiliations:** 1 Department of Anaesthesiology and Intensive Care, School of Medical Sciences, Universiti Sains Malaysia, Kelantan, Malaysia; 2 Department of Anaesthesiology and Intensive Care, Hospital Universiti Sains Malaysia, Kelantan, Malaysia; 3 Unit of Biostatistics and Research Methodology, School of Medical Sciences, Universiti Sains Malaysia, Kelantan, Malaysia

**Keywords:** meropenem failure, sepsis, outcomes, mortality, Intensive Care Unit

## Abstract

**Background:**

The aim of the study was to determine the predictors of meropenem treatment failure and mortality in the Intensive Care Unit (ICU).

**Methods:**

This was a retrospective study, involving sepsis and septic shock patients who were admitted to the ICU and received intravenous meropenem. Treatment failure is defined as evidence of non-resolved fever, non-reduced total white cell (TWC), non-reduced C-reactive protein (CRP), subsequent culture negative and death in ICU.

**Results:**

An Acute Physiology and Chronic Health Evaluation II (APACHE II) and duration of antibiotic treatment less than 5 days were associated with treatment failure with adjusted OR = 1.24 (95% CI: 1.15, 1.33; *P <* 0.001), OR = 65.43 (95% CI: 21.70, 197.23; *P <* 0.001). A higher risk of mortality was observed with higher APACHE and Sequential Organ Failure Assessment (SOFA) scores, initiating antibiotics > 72 h of sepsis, duration of antibiotic treatment less than 5 days and meropenem with renal adjustment dose with an adjusted OR = 1.21 (95% CI: 1.12, 1.30; *P* < 0.001), adjusted OR = 1.23 (95% CI: 1.08, 1.41; *P* < 0.001), adjusted OR = 6.38 (95% CI: 1.67, 24.50; *P* = 0.007), adjusted OR = 0.03 (95% CI: 0.01, 0.14; *P* < 0.001), adjusted OR = 0.30 (95% CI: 0.14, 0.64; *P =* 0.002).

**Conclusion:**

A total of 50 (14.12%) patients had a treatment failure with meropenem with 120 (48.02%) ICU mortality. The predictors of meropenem failure are higher APACHE score and shorter duration of meropenem treatment. The high APACHE, high SOFA score, initiating antibiotics more than 72 h of sepsis, shorter duration of treatment and meropenem with renal adjustment dose were predictors of mortality.

## Introduction

The antibiotic carbapenem is a broad-spectrum antimicrobial agent that counteracts many Gram-positive and Gram-negative aerobes and anaerobic organisms. Examples of carbapenem include imipenem, meropenem, ertapenem and doripenem. Imipenem was first introduced in 1987, followed by meropenem in 1996 (1). Pharmacologically, these different types are almost identical. They bind to penicillin-binding protein and cause bactericidal activity, eventually causing the lysis of the organism (1). All carbapenems demonstrate activity against extended-spectrum beta-lactamase enzymes produced by organisms aiming to inhibit the antibiotic (1).

Meropenem is formulated for intravenous administration at 1 g–2 g every 8 h due to poor absorption via the intestines. Thus, the most suitable usage is in Intensive Care Unit (ICU) settings for critically ill patients. In critically ill patients with septic shock, meropenem is prescribed as an empirical therapy due to good tissue penetration. It has been used widely for severe and potentially life-threatening infections, such as pneumonia, intra-abdominal infections, septicaemia, bacterial meningitis and febrile neutropenia (1). In comparison with meropenem, imipenem is used less due to the risk of seizure. Ertapenem is not suitable since most septic patients in the ICU suffer from low albumin. Therefore, less of the drug can act (2).

Meropenem is used against commonly Gram-negative organisms in the ICU, including *Pseudomonas aeruginosa, Acinetobacter baumannii* and *Klebsiella pneumoniae* (3). Meropenem is two to four times more effective than imipenem against Gram-negative organisms (3). However, imipenem is more potent than meropenem against Staphylococci, *Streptococcus* spp. and Enterococci (3). The most common pathogen that is resistant to meropenem is methicillin-resistant Staphylococci and *Enterococcus faecium* (4). Unfortunately, the common prescription of meropenem to patients has become a risk factor for the emergence of colonisation and infection with a meropenem-resistant pathogen, making such prescription no longer suitable (5). The use of meropenem with critically ill patients is necessary in our institution since most of the patients in our institution that arrive in septic shock exhibit multiorgan dysfunction. Inappropriately used carbapenem will increase carbapenem-resistant Enterobacteriaceae (CRE). The widespread carbapenemase production caused by Enterobacteriaceae was first reported in 2001 (6). Among Gram-negative bacteria, CRE is very difficult to treat, as it encodes the carbapenemase enzyme, which breaks down carbapenem anti-microbials, such as meropenem. Therefore, this study aims to determine the predictors of meropenem treatment failure and ICU mortality among patients receiving meropenem.

## Methods

### Setting and Study Design

This was a retrospective study at Universiti Sains Malaysia (USM) Hospital conducted from 1 January 2016 to 1 June 2019. Our institution has about 800 beds with 32 Level 3 (medical, surgical, neuro and trauma) adult ICU beds. USM Hospital is a teaching hospital in East Coast Peninsular Malaysia. The average yearly admission to the ICU is 1,600. Only the first course of treatment was included in the analysis. Using a two-proportion formula based on a previous study (9), a 95% confidence interval and 80% power, and after considering the 20% drop-out rate, the required sample was set at 47 patients for each group. The inclusion criteria selected sepsis and septic shock patients admitted to the ICU with ages equal to or more than 18 years old who received meropenem for at least 3 days. The cases were excluded if meropenem started as a prophylaxis, the patients had a CRE culture and they were admitted with a diagnosis of cardiogenic shock.

All prescriptions of meropenem were recorded on the pharmacy’s special form in our institution. Cases were identified from the drug order list provided by the pharmacy from 1 January 2016 to 1 June 2019. Patient demographic data, underlying diseases, clinical conditions, microbiology data and outcomes were recorded manually by primary investigators after reviewing the data of each eligible patient in the medical records library.

### Operational Definitions

#### Sepsis and Septic Shock

Sepsis is defined as an infection causing organ dysfunction due to a dysregulated host immune response (7). Septic shock means that sepsis patients have both persisting hypotension, which requires vasopressors to maintain a mean arterial pressure (MAP) > 65 mmHg and serum lactate levels > 2 mmol/L despite adequate volume resuscitation (7).

#### Treatment Success

In general treatment, success is defined by the resolution of fever, decreased TWCs and reduced C-reactive protein (CRP) levels 48 h after initiating meropenem (8–10). In our study, those who did not fulfill the criteria for treatment failure were considered treatment successes.

#### Treatment Failure

Clinical signs and symptoms and blood parameters commonly monitored in institutions to determine the success of treatment with meropenem include resolved fever, reduced TWCs, reduced CRP and subsequent negative culture. In our study, treatment failure was declared when a patient met all four criteria (unresolved fever, non-reduced TWCs and CRP, and a positive subsequent culture) despite being on meropenem.

It has been suggested that inotropic usage should be started early in septic shock cases to maintain a MAP of at least 65 mmHg and organ perfusion while waiting for the antibiotic to produce a response and the infection source to be controlled (11). The ideal course of treatment based on prior literature is to reduce inotropes as the targeted MAP is achieved (12, 13).

#### Prolonged Mechanical Ventilation

Prolonged mechanical ventilation is defined as patients requiring ventilators for more than 21 days for at least 6 h per day. Besides that, prolonged ventilation was defined as difficult to wean patients off the ventilator within 7 days of ventilation with up to three spontaneous breathing trials (14, 15). However, there were multifactorial leads to these circumstances. Thus, any reduction in ventilator days was defined as less than 21 days of required ventilation, including non-invasive ventilation post-extubation.

#### Prolonged ICU

Prolonged ICU stays have been defined as lasting more than 21 days (16). However, prolonged ICU stays of more than 14 days are associated with long-term mortality outcomes (17).

#### Reduced ICU

Reduced ICU stays were defined as less than 14 days in our study. There was a high mortality rate among septic shock patients; therefore, deaths from septic shock included patients in the ICU and hospital during admission (18, 19).

Acute Physiology and Chronic Health Evaluation II and Sequential Organ Failure Assessment

The severity of illness was based on an Acute Physiology and Chronic Health Evaluation II (APACHE II) and Sequential Organ Failure Assessment (SOFA) scores. In our study, patients were scored within 24 h to 48 h prior to starting meropenem treatment. APACHE II and SOFA scores are used to evaluate the functionality of several organ systems. The higher the score, the higher the mortality. APACHE II scores range from 0–71. SOFA scores range from 0–24.

#### Statistical Analysis

All data were analysed using IBM Statistical Package for the Social Sciences (SPSS), version 27.0 (IBM Corporation, Armonk, United Sates). Continuous variables were presented as means (with standard deviation [SD]) or medians (with interquartile range [IQR]) depending on the normality of the data distribution. Categorical data were presented as frequencies and percentages with *n* (%). Both descriptive and univariate statistical analyses were carried out where appropriate. Categorical data were tabulated using a contingency table and the Pearson’s chi-squared test was used to identify any potential associations between them. Subsequently, logistic regression analysis was adopted to determine the baseline characteristics that may have influenced meropenem treatment failure. Variables with *P*-values of less than 0.05 in the simple logistic regression were included in a stepwise (forward selection) multiple logistic regression. Hosmer-Lemeshow tests were applied to fit the model confirmation with values of more than 0.05. The relationships between each baseline characteristic and the dependent variable (treatment failure associated with meropenem) were evaluated. Variables that were found to be statistically significant and additional variables that might have plausibly affected the dependent variable were considered candidate variables in the multiple logistic regression. All probability values were two-sided and *P*-values of less than 0.05 were considered statistically significant.

## Results

### Demographic Data

There were 500 courses of meropenem therapy carried out during the period of study and 354 patients who fulfilled the inclusion and exclusion criteria were selected for record reviews. Based on the operational definitions, 50 cases were classified as treatment failures (14.12%) and 304 as treatment successes (85.88%). A total of 120 (48.02%) ICU deaths were recorded. Of the patients, 230 (64.9%) were male and 124 (35%) were female, with a mean age of 52.5 (SD = 17.7) years old. The surgical discipline contributed more, at 209 (59%), compared to the medical discipline. Of the patients, 331 (93.5%) were Malay, 22 (6.2%) were Chinese and 1 (0.3%) was Indian. Among all the selected patients, 288 (81.36%) had comorbidities, including hypertension (216, 61%), diabetes mellitus (175, 49.4%), chronic kidney disease (76, 21.5%), chronic lung disease (33, 9.3%), stroke (39, 11%), dyslipidaemia (100, 28.3%) and advanced cancer (42, 11.9%). The main diagnosis was hospital-acquired pneumonia (178, 50.28%), followed by community-acquired pneumonia (CAP) (72, 20.3%), intra-abdominal infection (54, 15.3%), necrotising fasciitis (29, 8.2%), urosepsis (12. 3.4%) and meningitis (9, 2.5%). The mean APACHE II and SOFA scores were 19.6 (SD = 5.9) and 6.3 (SD = 3.3), respectively. The proportion of cases in which meropenem was started early, after less than 72 h of sepsis, was 329 (92.9%), while it was started late in 25 (7.1%) patients. The number of patients who received meropenem for 5 days or more was 314 (88.7%). Most patients (272, 76.8%) received meropenem doses as loading doses combined with renal dosing in combination with a second antibiotic (Table 1). The biomarkers used to assess responses to meropenem therapy were temperature, TWC count and CRP (Table 2).

### Predictors of Meropenem Treatment Failure

The predictors of meropenem treatment failure among patients with septic shock according to the simple logistic regression were APACHE II and SOFA scores, chronic lung disease, source ward of admission to the ICU, and the time, dose and duration of initial meropenem administration. However, APACHE II scores, with a mean of 25.2 (SD 6.8), showed a significant association with meropenem treatment failure after multiple logistic regression, with an adjusted odd ratio (OR) of 1.24 (95% CI: 1.15, 1.33; *P* < 0.001). Similarly, the duration of antibiotic use exhibited significance, with an adjusted OR of 65.43 (95% CI: 21.70, 197.23; *P* < 0.001). Among the 50 patients experiencing meropenem treatment failure, 31 were prescribed this medication for 4–5 days (Table 3).

### Clinical Outcome of Meropenem Treatment Failure

Out of 50 patients, all had significantly prolonged ventilator days at χ^2^ = 15.4; df = 1; *P* < 0.001. Apart from that, 49 patients were unable to wean off inotropes at χ^2^ = 121.8; df = 1; *P* < 0.001. At the same time, 49 patients had no significant reduction in ICU stay at χ^2^ = 12.6; df = 1; *P* < 0.001. All 50 patients were found dead during their stay, either in ICU or in the ward during admission at χ^2^ = 63.0; df = 1; *P* < 0.001 (Table 4).

### Predictors of Mortality

A high risk of mortality was observed among patients with high APACHE scores (adjusted OR = 1.21; 95% CI: 1.12, 1.30; *P* < 0.001), high adjusted SOFA scores (OR = 1.23; 95% CI: 1.08, 1.41; *P* < 0.001) or whose meropenem doses were started after more than 72 h of sepsis (adjusted OR = 6.38; 95% CI: 1.67, 24.50; *P* < 0.007) as indicated in Table 5. On the other hand, a higher risk of mortality was observed among patients on antibiotics for less than 5 days (adjusted OR = 0.03; 95% CI: 0.01, 0.14; *P* < 0.001) and patients on adjusted renal meropenem (adjusted OR = 0.30; 95% CI: 0.14, 0.64; *P* < 0.002).

## Discussion

Treatment of healthcare-associated infection (HAI) and healthcare community-associated infection caused by Gram-negative organisms among ICU patients who present with septic shock and are given meropenem are common in our institution. Our institution is among the highest users of meropenem in Malaysia. With the emergence of multidrug organisms and the severity of patients in septic shock, the usage of a broad-spectrum antibiotic such as meropenem is often indicated in our institution. However, the usage of meropenem leads to the emergence of CRE, which poses a serious challenge. Therefore, our study aimed to explore the relevant factors in treatment failure and mortality following meropenem therapy so that we could control the widespread usage of meropenem. A lack of source control and inadequate antibiotics are some of the factors (9, 19). At this point, there is no fixed definition of individual antibiotic treatment failure.

The baseline ages were similar in our study population in cases of treatment success and failure, at means of 52.13 (SD17.65) and 54.70 (SD17.87) years old. The main disciplines in which meropenem was prescribed in our institution were surgical and medical, while the main diagnosis for prescription was HAI.

The heterogeneity of patients admitted to ICU, such as their differences in age, severity of illness, source of infection and comorbidities (20), posed a challenge to determining the factors associated with meropenem treatment failure and mortality. Our study found that higher APACHE II scores and durations of treatment of less than 5 days were independently associated with treatment failure. The observed predictors of mortality were higher APACHE and SOFA scores, initiation of antibiotics after more than 72 h of sepsis, a duration of antibiotic treatment of less than 5 days and a dose of meropenem with renal adjustment.

### Acute Physiology and Chronic Health Evaluation II Score

An APACHE II score consists of all variables measured in critically ill patients within 24 h–28 h of admission. The APACHE II score is based on 12 physiological measurements, age and chronic health conditions. Our study revealed that higher APACHE II scores were associated with treatment failure. A high APACHE II score is known to be associated with mortality when antibiotics are used against *Acinetobacter* infections in most ICU studies (21, 22). However, the use of APACHE II scores of surgical patients to predict treatment failure and triage patients is unacceptable. The decision to triage patients must be based on clinical judgment rather than single parameters such as APACHE II scores (23). Their use in neurosurgical patients is also inferior for predicting in-hospital mortality (24).

### Duration of Treatment

Old guidelines stated that before discontinuing antibiotics for CAP, there must be an improvement in clinical symptoms, such as fever resolution, after 48 h–72 h and no clinical CAP symptoms (25). A study revealed that only 42% of patients treated empirically with meropenem for nosocomial infections undergo de-escalation therapy (26). The main reason for not de-escalating is a lack of microbiological evidence and an abdominal source of infection (26). In our institution, especially in complicated intrabdominal infections, it is difficult to determine which polymicrobial organisms are colonisers and which are true pathogens. Therefore, de-escalation was difficult in our cohort of septic patients. Moreover, the prevalence of superbugs was high in our critically ill patients. The latest sepsis guidelines recommend 7 days–10 days of antibiotics for treating patients with sepsis and septic shock (27). The recommended duration of antibiotics for nosocomial pneumonia is 7 days or less (28), while it is 5 days for CAP (29), 4 days for intrabdominal infection with source control (30) and 7 days for acute pyelonephritis (31).

### Sequential Organ Failure Assessment

A SOFA score above 12 (OR = 6.8; 95% CI: 1.3, 37; *P* = 0.025) has been identified as a risk factor for death in septic shock patients (32). In another study, a reduction in SOFA score of more than 25% on day 7 was one of the predictors of 28-day mortality (32). A day-3 change in SOFA score reveals an area under the receiver operating characteristic curve (AUROC) of 0.68 (95% CI: 056, 0.79) (33). A 50% decrease in SOFA was associated with 61.3% sensitivity and an 85.9% negative predictive value for ICU mortality (34). In another study involving ventilator-associated pneumonia with prolonged mechanical ventilation, SOFA score was one of the predictive outcomes of 30-day mortality, with a hazard ratio (HR) of 1.30 (95% CI: 1.12, 1.52; *P* < 0.001) (35). Meropenem was formerly used in our institution in certain patients with carbapenem-resistant *Acinetobacter baumannii* (CRAB) in combination with high-dose sulbactam. The predictor of 14-day mortality in CRAB was the severity of illness (OR = 1.38 for each 1-point increase in SOFA score; 95% CI: 1.21, 1.56) (36). Our study also found that SOFA score is one of the predictors of mortality.

### Timing of Initiation of Antibiotics

Guidelines for surviving sepsis or septic shock recommend that antibiotics need be initiated within 1 h of diagnosis (37). However, in our institution, there is limited access to the initiation of early broad-spectrum antibiotics in septic shock patients. Our institution’s antibiotic stewardship policy does not encourage prescribers to initiate meropenem as one of the initial antibiotics in the emergency department (ED) unless indicated. Therefore, most of our cohort patients received meropenem more than 72 h after the diagnosis of sepsis. Most of the time, by 72 h, the patient was already in the ICU. In the ED, our institution usually prescribes piperacillin-tazobactam for septic shock and escalates to meropenem if indicated in the ICU. For patients with presumed sepsis or septic shock, the administration of each antibiotic ordered should be initiated promptly, with healthcare systems working to reduce that time to as short a duration as feasible (38). Early diagnosis is crucial to avoid delays in starting appropriate antibiotics (39). Delays in antibiotics are associated with increased mortality (40). Our study revealed that late initiation of meropenem was associated with treatment failure.

### Dose of Antibiotics

Our study revealed that dose adjustment in renal failure is one of the predictors of meropenem treatment failure. The precise dose of antibiotics in the ICU for all critically ill patients is still a subject of research. The dosage viewed as correct in many national and international guidelines is based on normal and volunteer patient studies. Therefore, the recommended concentration of antibiotics in critically ill patients may not be accurate due to differences in the volume of distribution, clearance and organ function, such as kidney and liver, which are the main organs for metabolising and excreting antibiotics. Various factors, such as serum albumin, fluid status, systemic inflammatory response syndrome, microvascular failure, and changes in renal and hepatic function may change the pharmacokinetics, which can alter the concentration of the antibiotic in critically ill patients (41). Therefore, antibiotics should be individualised in the future. In general, most critically ill patients fail to attain the desired concentration of 50% free time (*f*T) above the minimum inhibitory concentration (MIC). Roberts et al. (42) found there was an association increasing 100% *f*T > MIC ratio (OR = 1.53; *P* = 0.03) and positive clinical outcome. Fluid resuscitation in the ED and ICU and capillary leaks due to septic shock could be some of the factors in subtherapeutic antibiotic concentrations in critically ill patients (43). Other factors, such as acute kidney injury and augmented renal clearance, also play an important role in altering levels of antibiotics (44). The other factor is that the current dose used in the ICU is not guided by the MIC of bacteria-resistant organisms (45). The initial 24 h–48 h of high-dose meropenem may be needed to overcome this subtherapeutic concentration in septic shock patients (46, 47).

## Conclusion

The predictors of meropenem treatment failure among patients with septic shock in the ICU were high APACHE II scores and a treatment duration of less than 5 days. The predictors of mortality were high APACHE and SOFA scores, a sepsis duration of 72 h prior to initiation of meropenem, a duration of treatment of less than 5 days and patients receiving meropenem with renal adjustment.

## Limitations

This study was limited to a single centre, with patient management based on the presiding doctor’s clinical judgement and no comparison with the decisions of other centres. We also did not include data on the source control of infections, prescription changes and escalation of care, which are important factors contributing to antibiotic failure. The data were collected by only one researcher, with no countercheck by anyone else. Antibiotic prescriptions in our institution are still manual as opposed to electronic systems. There were significant variations in definitions of treatment failure in a systematic review and narrative synthesis reported in 2022 (48).

## Figures and Tables

**Table 1:** Characteristics of participants according to treatment outcome

Variables	Success (*n* = 304)	Failure (*n* = 50)

	*n* (%)	*n* (%)
Age (years old), mean (SD)	52.13 (17.65)	54.70 (17.87)
Gender		
Female	103 (83.1)	21 (16.9)
Male	201 (87.4)	29 (12.6)
Ethnicity		
Malay	284 (85.8)	47 (14.2)
Chinese	19 (86.4)	3 (13.6)
Others	1 (100.0)	0 (0.0)
APACHE score, mean (SD)	18.67 (5.23)	25.22 (6.85)
SOFA score, mean (SD)	5.90 (2.74)	8.74 (4.82)
Comorbidity		
Hypertension	186 (86.1)	30 (13.9)
Diabetes	153 (87.4)	22 (12.6)
Chronic kidney disease	62 (81.6)	14 (18.4)
Chronic lung disease	23 (69.7)	10 (30.3)
Stroke	31 (79.5)	8 (20.5)
Dyslipidaemia	87 (87.0)	13 (13.0)
Advanced cancer	36 (85.7)	6 (14.3)
Discipline		
Medical	119 (82.1)	26 (17.9)
Surgical	185 (88.5)	24 (11.5)
Source of admission		
Operating theatre	125 (92.6)	10 (7.4)
Emergency department	43 (70.5)	18 (29.5)
Ward	136 (86.1)	22 (13.9)
Time of antibiotic started		
≤ 72 h of sepsis	287 (87.2)	42 (12.8)
> 72 h of sepsis	17 (68.0)	8 (32.0)
Duration of antibiotics		
< 5 days	9 (22.5)	31 (77.5)
≥ 5 days	295 (93.9)	19 (6.1)
Types of meropenem treatment		
Loading + Renal adjustment + Combination therapy	231 (84.9)	41 (15.1)
Loading + Combination	72 (91.1)	7 (8.9)
Loading + Monotherapy	1 (33.3)	2 (66.7)

**Table 2:** Clinical outcome of meropenem therapy

Outcome	Success (*n* = 304) *n* (%)	Failure (*n* = 50) *n* (%)
Fever		
Not resolved	10 (16.7)	50 (83.3)
Resolved	294 (100.0)	0 (0.0)
TWC		
Not reduced	93 (65.0)	50 (35.0)
Reduced	211 (100.0)	0 (0.0)
CRP		
Not reduced	139 (73.5)	50 (26.5)
Reduced	165 (100.0)	0 (0.0)

Notes: TWC = total white cell; CRP = C-reactive protein

**Table 3:** Predictors for meropenem treatment failure

	Crude b	Crude OR (95% CI)	*P*-value	Adj. b	Adj. OR (95% CI)	*P*-value
Age, years old	0.01	1.01 (0.99, 1.03)	0.342			
Gender						
Female	0	1	0.266			
Male	−0.35	0.71 (0.39, 1.30)				
Ethnicity						
Malay	0	1				
Chinese	−0.05	0.95 (0.27, 3.35)	0.942			
APACHE score	0.18	1.20 (1.14, 1.27)	< 0.001	0.22	1.24 (1.15, 1.33)	< 0.001
SOFA score	0.23	1.25 (1.15, 1.37)	< 0.001			
Comorbidity						
Hypertension	−0.06	0.94 (0.51, 1.74)	0.852			
Diabetes	−0.25	0.78 (0.43, 1.42)	0.408			
Chronic kidney disease	0.42	1.52 (0.77, 2.99)	0.227			
Chronic lung disease	−1.12	0.33 (0.15, 0.74)	0.007			
Stroke	0.52	1.68 (0.72, 3.90)	0.229			
Dyslipidaemia	−0.11	0.90 (0.46, 1.78)	0.763			
Advanced cancer	0.02	1.02 (0.40, 2.55)	0.974			
Discipline						
Medical	0	1				
Surgical	−0.52	0.59 (0.33, 1.08)	0.089			
Source of admission						
Operating theatre	0	1				
Emergency department	1.66	5.23 (2.24, 12.21)	< 0.001			
Ward	0.70	2.02 (0.92, 4.44)	0.079			
Time of antibiotic started						
≤ 72 h of sepsis	0	1				
> 72 of sepsis	1.17	3.22 (1.31, 7.91)	0.011			
Age, years old	0.01	1.01 (0.99, 1.03)	0.342			
Duration of antibiotics						
< 5 days	0	1		0		
≥ 5 days	−3.98	0.02 (0.01, 0.05)	< 0.001	−4.42	65.43 (21.70, 197.23)	< 0.001
Types of meropenem treatment						
Loading + Renal adjustment + Combination therapy	0	1				
Loading + Combination/Monotherapy	−0.36	0.70 (0.32, 1.50)	0.352			

Notes: Variable race: One patient is other race and excluded from analysis. Variable types of meropenem treatment: Loading + Monotherapy is being combined with Loading + Combination therapy. Forward LR method of variable selection was applied. Hosmer-Lemeshow test χ^2^(8) = 5.99, *P* = 0.648; Classification table overall percentage correct = 92.9%; AUROC = 92.1 % (95% CI: 87.0, 97.2).

Summary: APACHE score and duration of antibiotics were the independent predictors for meropenem treatment failure. Patients with higher APACHE score had increased risk for meropenem failure (adjusted OR = 0.22; 95% CI: 1.15, 1.33; *P* < 0.001). Similarly, patients on antibiotics for at least 5 days had lower risk for meropenem failure (adjusted OR = 0.01; 95% CI: 0.004, 0.04; *P* < 0.001)

**Table 4:** Clinical outcome of patients treated with meropenem

Outcome	Success (*n* = 304) *n* (%)	Failure (*n* = 50) *n* (%)	χ^2^ (df)	*P*-value
Reduced ionotropic support				
No	61 (55.5)	49 (44.5)	121.8 (1)	< 0.001
Yes	243 (99.6)	1 (0.4)		
Reduced ventilator days				
No	230 (82.1)	50 (17.9)	15.4 (1)	< 0.001
Yes	74 (100.0)	0 (0.0)		
Reduced ICU stay				
No	231 (92.5)	49 (7.5)	12.6 (1)	< 0.001
Yes	73 (98.6)	1 (1.4)		
In hospital mortality				
Alive	184 (100.0)	0 (0.0)	63.0 (1)	< 0.001
Dead	120 (70.6)	50 (29.4)		

**Table 5:** Determinants of mortality among patients treated with meropenem

	Crude b	Crude OR (95% CI)	*P*-value	Adj. b	Adj. OR (95% CI)	*P*-value
Age, years old	0.02	1.02 (1.01, 1.03)	0.005			
Gender						
Female	0	1				
Male	−0.22	0.80 (0.52, 1.24)	0.321			
Ethnicity						
Malay	0	1				
Chinese	0.68	1.96 (0.80, 4.81)	0.140			
APACHE score	0.27	1.31 (1.23, 1.40)	< 0.001	0.19	1.21 (1.12, 1.30)	< 0.001
SOFA score	0.39	1.48 (1.34, 1.64)	< 0.001	0.21	1.23 (1.08, 1.41)	< 0.002
Comorbidity						
Hypertension	0.51	1.67 (1.08, 2.57)	0.021			
Diabetes	0.55	1.72 (1.13, 2.63)	0.011			
Chronic kidney disease	0.93	2.53 (1.49, 4.30)	0.001			
Chronic lung disease	1.16	3.20 (1.44, 7.09)	0.004			
Stroke	0.62	1.85 (0.94, 3.67)	0.077			
Dyslipidaemia	0.57	1.77 (1.11, 2.82)	0.017			
Advanced cancer	0.42	1.52 (0.79, 2.91)	0.210			
Discipline						
Medical	0	1				
Surgical	−0.73	0.48 (0.32, 0.74)	0.001			
Source of admission						
Operating theatre	0	1				
Emergency department	1.01	2.72 (1.46, 5.08)	0.002			
Ward	0.55	1.73 (1.09, 2.77)	0.021			
Time of antibiotic started						
≤ 72 h	0	1		0	1	
> 72 h	1.85	6.34 (2.13, 18.88)	0.001	1.85	6.38 (1.67, 24.50)	0.007
Duration of antibiotics						
< 5 days	0	1		0	1	
≥ 5 days	−2.82	0.06 (0.02, 0.20)	< 0.001	−3.40	0.03 (0.01, 0.14)	< 0.001
Types of meropenem treatment						
Loading + Renal adjustment + Combination therapy	0	1		0	1	
Loading + Combination / Monotherapy	−1.59	0.20 (0.11, 0.37)	< 0.001	−1.22	0.30 (0.14, 0.64)	0.002

Notes: Forward LR method of variable selection was applied. Hosmer-Lemeshow test χ^2^(8) = 7.12, *P* = 0.521; Classification table overall percentage correct = 81.4%; AUROC = 87.1% (95% CI: 83.5, 90.8).

Summary: Multiple logistic regression analysis reveals that the APACHE score, SOFA score, time of initiation of antibiotics, duration of antibiotics and types of meropenem treatment the independent predictors for mortality among patients treated with meropenem
